# Phage Revolution Against Multidrug-Resistant Clinical Pathogens in Southeast Asia

**DOI:** 10.3389/fmicb.2022.820572

**Published:** 2022-01-27

**Authors:** Mark B. Carascal, Donna May dela Cruz-Papa, Roland Remenyi, Mely Cherrylynne B. Cruz, Raul V. Destura

**Affiliations:** ^1^Clinical and Translational Research Institute, The Medical City, Pasig, Philippines; ^2^Institute of Biology, College of Science, University of the Philippines Diliman, Quezon City, Philippines; ^3^Department of Biological Sciences, College of Science, University of Santo Tomas, Manila, Philippines; ^4^Research Center for Natural and Applied Sciences, University of Santo Tomas, Manila, Philippines; ^5^The Graduate School, University of Santo Tomas, Manila, Philippines; ^6^National Institutes of Health, University of the Philippines Manila, Manila, Philippines

**Keywords:** bacteriophage, multidrug resistance, phage revolution, phage therapy, Southeast Asia

## Abstract

Southeast Asia (SEA) can be considered a hotspot of antimicrobial resistance (AMR) worldwide. As recent surveillance efforts in the region reported the emergence of multidrug-resistant (MDR) pathogens, the pursuit of therapeutic alternatives against AMR becomes a matter of utmost importance. Phage therapy, or the use of bacterial viruses called bacteriophages to kill bacterial pathogens, is among the standout therapeutic prospects. This narrative review highlights the current understanding of phages and strategies for a phage revolution in SEA. We define phage revolution as the radical use of phage therapy in infectious disease treatment against MDR infections, considering the scientific and regulatory standpoints of the region. We present a three-phase strategy to encourage a phage revolution in the SEA clinical setting, which involves: (1) enhancing phage discovery and characterization efforts, (2) creating and implementing laboratory protocols and clinical guidelines for the evaluation of phage activity, and (3) adapting regulatory standards for therapeutic phage formulations. We hope that this review will open avenues for scientific and policy-based discussions on phage therapy in SEA and eventually lead the way to its fullest potential in countering the threat of MDR pathogens in the region and worldwide.

## Introduction

A recent report identified Southeast Asia (SEA) as a global epicenter of antimicrobial resistance and emerging infectious diseases evolution (Chua et al., 2021). In the report by Chua et al. (2021) and in our paper, SEA is defined as the region that is home to the member states of the Association of Southeast Asian Nations (ASEAN; composed of Brunei, Cambodia, Indonesia, Laos, Malaysia, Myanmar, Philippines, Singapore, Thailand, and Vietnam). The issue of multidrug resistance (MDR) remains one of the region’s substantial challenges. Over the last 10 years, clinicians and researchers reported the rise of critical MDR organisms in SEA (Table 1). Alarming reports from the region also point to the emergence of resistance to colistin, a last-resource antibiotic, making MDR a concern of global interest and high priority. The current burden of AMR in the region remains unknown. Still, country-specific reports indicated high percentages of carbapenem-resistant *Acinetobacter baumannii* (39–68%), *Pseudomonas aeruginosa* (15–44%), and *Klebsiella pneumoniae* (4–29%) in Malaysia, Philippines, Thailand, and Vietnam (Center for Disease Dynamics, Economics, and Policy, 2021). Unregulated access to antibiotics, weak antibiotic stewardship programs, and low-quality antibiotics are the primary culprits of rampant MDR in SEA (Zellweger et al., 2017). Ultimately, the decreasing efficacy of our existing antibiotics and the shortage of newly discovered antimicrobials in the global pipeline will exacerbate the current situation of MDR. The World Health Organization estimates that antibiotic resistance will cause 10 million deaths in 2050, nearly half of which coming from Asia-Pacific, including SEA (World Health organization, 2019).

**Table 1:** Reports of critical multidrug-resistant (MDR) organisms from clinical isolates in Southeast Asia (SEA) from 2011 to 2021.

Critical MDR organisms^1^	Reporting SEA country	References
Carbapenem-resistant *Acinetobacter baumannii*	Cambodia	Vlieghe et al., 2013
	Indonesia	Karuniawati et al., 2013; Saharman et al., 2018, 2021
	Malaysia	Kim et al., 2013; Biglari et al., 2015, 2017
	Philippines	Velasco et al., 2020b
	Singapore	Koh et al., 2012; Kim et al., 2013; Blackwell et al., 2017; Ng et al., 2018
	Thailand	Teo et al., 2015
	Vietnam	Nhu et al., 2014
Extended-spectrum beta-lactamase (ESBL)-producing, carbapenem-resistant *Enterobacteriaceae*	Indonesia	Karuniawati et al., 2013
	Malaysia	Zainol Abidin et al., 2015
	Philippines	Sheng et al., 2013; Chou et al., 2016; Velasco et al., 2017
	Singapore	Koh et al., 2012; Sheng et al., 2013
	Thailand	Rimrang et al., 2012; Sheng et al., 2013
	Vietnam	Sheng et al., 2013; Biedenbach et al., 2014
Carbapenem-resistant *Pseudomonas aeruginosa*	Malaysia	Liew et al., 2018
	Singapore	Koh et al., 2012
	Thailand	Khuntayaporn et al., 2012, 2013, 2019
Colistin-resistant Gram-negative pathogens	Cambodia	Ström Hallenberg et al., 2019
	Indonesia	Rahayuningtyas et al., 2020
	Laos	Hadjadj et al., 2019
	Malaysia	Mobasseri et al., 2019; Aklilu and Raman, 2020
	Myanmar	San et al., 2019
	Philippines	Velasco et al., 2020a
	Singapore	Teo et al., 2016; La et al., 2019
	Thailand	Eiamphungporn et al., 2018
	Vietnam	Berglund et al., 2018; Yamamoto et al., 2019

1 Critical MDR organisms based on the World Health Organization (2017), except for colistin-resistant Gram-negative pathogens.

The threat of an imminent “post-antibiotic” era pushed scientists to identify novel agents and therapeutic approaches to combat MDR organisms. Harper and Enright (2011) described this situation where researchers progressively look for new antimicrobial agents or revisit traditional therapeutic options as a “perfect storm.” One of the therapeutic prospects to mitigate the AMR crisis is phage therapy. Phage therapy refers to the use of bacterial viruses, called bacteriophages (or simply phages), to treat and control infections. Phage therapy harnesses the capabilities of the virus to specifically bind and inject their genetic material into their target bacteria, produce progeny virions, and eventually lyse their host. Although the pioneering works of d’Herelle (1917) and Twort (1915) on phage therapy sparked the scientific community’s interest in its prospects for therapeutic application against human and animal infections, it was quickly overshadowed by the discovery and extensive use of penicillin. Members of the medical community continued using phages to manage common infections until the late 1940s, with applications reported in Europe and the United States (Harper and Enright, 2011; Ghannad and Mohammadi, 2012; Jin et al., 2012; Barbu et al., 2016). In recent years, a rekindled interest in phage therapy for treating MDR organisms led to remarkable breakthroughs. While several recent review articles highlight the advances of phage therapy (Khalid et al., 2021; Ng et al., 2021; Wu and Zhu, 2021), none of these papers focus on the ongoing research and practical applicability in SEA, particularly in the clinical setting. A recent perspective paper discussed phage applications in SEA, focusing on livestock, aquaculture, and agriculture sectors (Rajandas et al., 2021). The potential of phage therapy for clinical applications, however, is not examined. In this narrative review of the published literature, we assess the current understanding of phages, challenges in phage therapy, and strategies that will pave the way for a phage revolution, which we define as the use of phage therapy in infectious disease treatment in humans, considering the scientific and regulatory standpoint in SEA. Asavarut and Hajitou (2014) loosely mentioned “phage revolution” in a book review on phage therapy. In our article, we expound on the concept of phage revolution by proposing a three-phase strategy that can guide member states of the ASEAN region. The proposed roadmap is essential for realizing phage therapy’s promise in countering the threat of MDR clinical pathogens in the region and potentially in the world.

## Phages in SEA

Phages are viral particles composed of genetic material enclosed in a capsid or head, and in most cases, a proteinaceous tail. The term “bacteriophage” literally translates to “bacteria-eater.” Biologists describe them as natural parasites (or predators) of bacteria and associate them with maintaining the balance of microorganisms on Earth (Wittebole et al., 2014). Tailed phages of the class *Caudoviricetes* including podovirus, myovirus, and siphovirus (Liu et al., 2021), and the polyhedral *Microviridae* family are usually associated with phage therapy applications (Supplementary Figure 1; Harper and Enright, 2011; Lin et al., 2017). Scientists attribute the phages’ antibacterial activity to their well-established life cycle (lytic or lysogenic; Supplementary Figure 2). In some cases, phages can adapt both lysogenic and lytic strategies (i.e., pseudolysogeny) as alternative infection steps in response to different host strains, host physiology, and environmental changes (Mäntynen et al., 2021). Since phages are ubiquitous, it is not surprising that they may be isolated from an extensive range of sampling sites like feces, seawater, sewage, soil, sludge, and anywhere bacteria may grow (Khawaja et al., 2016; Bhetwal et al., 2017).

In the past decade, scientists reported the isolation of diverse phages in SEA from different sample types, with potential activities against human pathogens (Table 2). Among the phages discovered in the region with notable activity against MDR organisms are: (1) phages vB_AbaM_PhT2 (Styles et al., 2020) and AB1801 (Wintachai et al., 2019), which exhibited anti-MDR *A. baumannii* activity, *in vitro* and *in vivo*, respectively, (2) phage UPM2146 (Assafiri et al., 2021), which exhibited activity against carbapenem-resistant *K. pneumoniae*, *in vitro* and *in vivo*, (3) phage C34 (Guang-Han et al., 2016), which controlled naturally resistant *Burkholderia pseudomallei* bacterial load, *in vivo*, (4) phage ΦHN10 (Phothichaisri et al., 2018), which exhibited the highest breadth of activity against various *Clostridiodes difficile* strains, *in vitro*, (5) phage ΦKAZ14 (Ahmad et al., 2015), which induced lysis of extended-spectrum beta-lactamase (ESBL)-producing *Escherichia coli*, *in vitro*, (6) phages UPMK_1 and 2 (Dakheel et al., 2019) and ΦNUSA-1 and 10 (Tan et al., 2020), which all exhibited lytic activity against methicillin-resistant *Staphylococcus aureus* (MRSA), and anti-biofilm activity for the first two phages in *in vitro* experiments, (7) cocktail of phages vB_SenS_WP109, WP110, and WP128 (Pelyuntha et al., 2021), which induced lysis of multiple MDR *Salmonella* serovars, *in vitro*, and (8) phages KP1, 2 (Cornista et al., 2019), and KP1801 (Wintachai et al., 2020), which were shown to infect ESBL-producing *K. pneumoniae*, *in vitro*. Although the actual diversity of phages remains understudied in SEA, we believe that there is a significant richness of phage strains in this region, a renowned biodiversity hotspot not only in terms of fauna and flora but also in microbiota. This hypothesis is supported by the growing number of genome data entries for phage in the ASEAN Microbial Database (AMIBASE), with more than 200 recorded distinct phage entries, with each entry containing one or more phage strains or genomes (https://www.amibase.org/index.php, accessed on 20 December 2021). The majority of the discovered phages belong to the class *Caudoviricetes* (188 records), dominated by siphovirus (80 records) and myovirus (60 records). *Microviridae* phages were also reported, although infrequently (four records). Clinically important host ranges of the reported phages include *Acinetobacter*, *Aeromonas*, *Bacillus*, *Bordetella*, *Brucella*, *Burkholderia*, *Campylobacter*, *Caulobacter*, *Citrobacter*, *Clostridioides*, *Corynebacterium*, *Edwardsiella*, *Enterobacter*, *Enterococcus*, *Erysipelothrix*, *Escherichia*, *Helicobacter*, *Leptospira*, *Listeria*, *Mycobacterium*, *Nocardia*, *Propionibacterium*, *Pseudomonas*, *Raoultella*, *Salmonella*, *Serratia*, *Shewanella*, *Shigella*, *Staphylococcus*, *Stenotrophomonas*, *Streptococcus*, *Vibrio*, *Weissella*, and *Yersinia*. However, most of the phage’s actual activities against these hosts have yet to be described. Phage sources listed in AMIBASE include freshwater, wastewater, sludge, marine water and sediment, soil, hot spring, bioreactor, humans, plants, invertebrates, and air, although the isolation procedures were not specified. Singapore contributed most of the records (191), followed by Malaysia (187) and Thailand (171). Brunei, Laos, and Myanmar have no phage records in AMIBASE as of this writing, indicating potential avenues for discovering novel phages in these countries. We hope that through this initial report, we can increase the number of phages and phage genomes annotated in the region so more data on phages can be shared and studied by SEA researchers.

**Table 2:** Phages with potential biomedical applications reported in SEA from 2011 to 2021.

Phage/Phage name	Host	Source	Reporting SEA country	References
*Acinetobacter* phages (AB1801, vB_AbaM_PhT2)	*Acinetobacter baumannii*	Hospital wastewater	Thailand	Wintachai et al., 2019; Styles et al., 2020
*Aeromonas* phages (UP87, AecaKS148, phage 2/5, B614, TG25P/CT45P, PVN02)	*Aeromonas* spp. (*A. hydrophila*, *A. salmonicida*, *A. caviae*)	Sewage, freshwater	Philippines, Thailand, Vietnam	Dela Cruz-Papa et al., 2014, 2017; Wangkahad et al., 2015; Le et al., 2018; Hoang et al., 2019; Tu et al., 2020
*Burkholderia* phages (Phage C34)	*Burkholderia pseudomallei*	Seawater, soil	Malaysia, Thailand	Shan et al., 2014; Guang-Han et al., 2016; Withatanung et al., 2016
*Clostridioides* phages (ΦHR24, ΦHN10, ΦHN16-1, ΦHN16-2, ΦHN50)	*Clostridioides difficile* (formerly *Clostridium difficile*)	Clinical isolates (induced)^1^	Thailand	Phothichaisri et al., 2018
Coliphages (ØEC1, EC1-UPM, ΦKAZ14, YD-2008, CS EPEC, BL EHEC, BI-EHEC)	Coliforms (i.e., enteropathogenic/Enterohemorrhagic *Escherichia coli*)	Poultry and farm feces, urban catchment, tissue samples	Indonesia, Malaysia, Singapore	Lau et al., 2012; Gan et al., 2013; Rezaeinejad et al., 2014; Ahmad et al., 2015; Vergara et al., 2015; Sellvam et al., 2018; Lukman et al., 2020; Dewanggana et al., 2021; Sjahriani et al., 2021; Waturangi et al., 2021
*Edwardsiella* phages (MK7)	*Edwardsiella ictaluri*	Tissue samples	Vietnam	Hoang et al., 2018
*Enterobacter* phages (EnspKS513, EspM4VN)	*Enterobacter* sp.	Sewage, freshwater, soil	Thailand, Vietnam	Wangkahad et al., 2015; Thanh et al., 2020
*Enterococcus* phages (AIM06, SR14)	*Enterococcus faecalis*	Watershed	Thailand	Chyerochana et al., 2020
*Klebsiella* phages (KlpnKS648, KP1801, UPM2146)	*Klebsiella pneumoniae*	Sewage, Hospital waste, freshwater	Malaysia, Thailand	Wangkahad et al., 2015; Cornista et al., 2019; Wintachai et al., 2020; Assafiri et al., 2021
*Lactococcus* phages (PLgT-1, PLgY-30)	*Lactococcus gervieae*	Tissue isolates (induced)^1^	Vietnam	Hoai et al., 2016, 2019
*Listeria* phages (LP019, LP040, LP041)	*Listeria monocytogenes*	Seafood processing environment	Thailand	Vongkamjan et al., 2017; Vu et al., 2021
*Proteus* phages (pPM_01)	*Proteus mirabilis*	Sewage	Malaysia	Wirjon et al., 2016
*Salmonella* phages (Φst1, ST-W77, SE-W109, vB_SenS_WP109, vB_SenS_WP110, vB_SenP_WP128)	*Salmonella enterica*	Dairy farm, poultry, clinical samples	Malaysia, Thailand	Wong et al., 2014; Wongsuntornpoj et al., 2014; Phothaworn et al., 2019, 2020; Pelyuntha et al., 2021
*Shigella*				
*Staphylococcus* phages (UPMK_1, UPMK_2, ΦNUSA-1, ΦNUSA-10)	*Staphylococcus aureus*	Sewage, seawater, meats	Malaysia	Dakheel et al., 2019; Tan et al., 2020
*Vibrio* phages (VPUSM 1-11, PSU2598, PSU4118, PSU 4211, seahorse, HY01)	*Vibrio* spp. (*V. alginolyticus*, *V. campbellii, V. cholerae*, *V. harveyi*, *V. parahaemolyticus*)	Freshwater, sewage, shellfish, marine sediment	Malaysia	Al-Fendi et al., 2014; Yingkajorn et al., 2014; Lal et al., 2016a,b, 2017; Thammatinna et al., 2020; Nuidate et al., 2021
*Weisella* phage (Φ22, PWc)	*Weisella* spp. (*W. ceti*, *W. cibaria*)	Fermented meat, tissue samples	Thailand, Vietnam	Pringsulaka et al., 2011; Hoai et al., 2018

1 Temperate phage induced *via* mitomycin C.

## Phage Research in SEA

**Figure 1:**
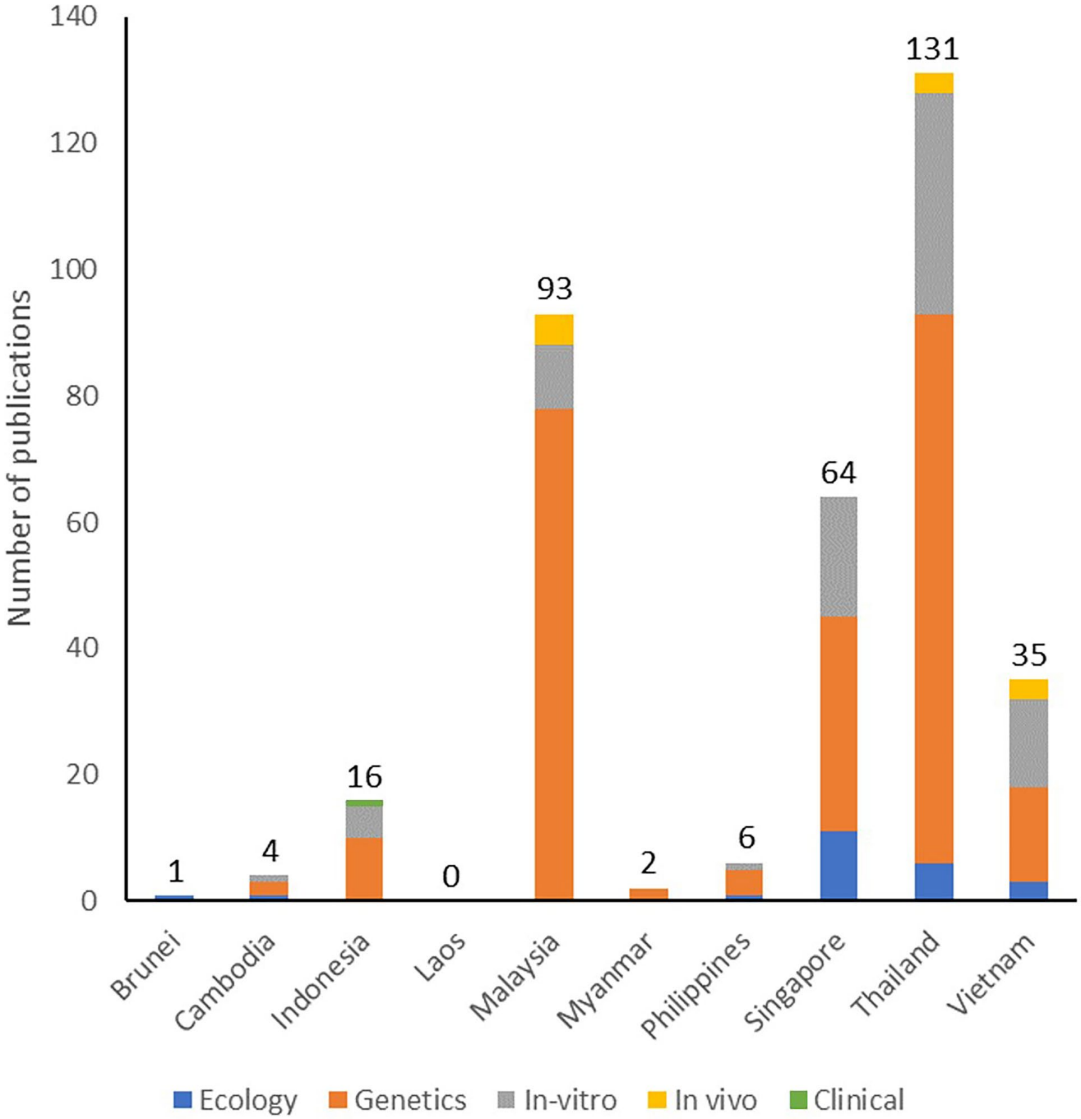
Number and types of original research articles on phages published in SEA and indexed in PubMed from 2011 to 2021.

Our understanding of phages and phage therapy relies on the research of basic and applied scientists worldwide. To determine the extent and coverage of biomedical phage research in SEA, we conducted a preliminary original article search in PubMed (https://www.pubmed.ncbi.nlm.nih.gov/, accessed 20 December 2021) using the keywords “phage” AND “[ASEAN country]” from 2011 to 2021. We then screened the articles and only included original research conducted in SEA countries or those which used samples coming from SEA. We attempted a localized biomedical literature search but failed due to the unavailability of a health research database in most SEA countries. In the Philippines (HERDIN, https://www.registry.healthresearch.ph, accessed 01 July 2021), Malaysia (Ministry of Health Virtual Library, https://www.vlib.moh.gov.my, accessed 01 July 2021), and Indonesia (https://www.neliti.com, accessed 01 July 2021), the available health research registries reported no local biomedical publications on phages. We summarized our literature search result in Figure 1. The current literature search we conducted is limited only to PubMed, but it can help determine the region’s strengths and priorities on biomedical phage research. Thailand, Malaysia, and Singapore were the top three contributors to biomedical-related phage research in SEA. In the latest economic report, these countries have high gross domestic product (Vu, 2020) which may reflect their capability to perform high-end scientific explorations for potential high-value therapeutics, such as phage therapy. Hence, it is not surprising to see these countries as the frontrunners for phage research in the region. We believe that the studies on phages in SEA can still be increased, particularly by focusing on its applications against MDR organisms. By leveraging the existing phage expertise in the region, a research consortium on phages could be created to further address the research gaps on phages in SEA. We will expound on this concept later on in this review.

Most research on phages in SEA consists of genetic studies, including genomic characterization of phages and bioengineering applications, such as phage typing and phage display. In terms of bioengineering phages for therapeutic applications, SEA scientists reported the use of phages for targeted gene therapy (Namdee et al., 2018; Przystal et al., 2019; Chongchai et al., 2021) and adaptation of nanomaterial-based phage delivery systems (Adamu Ahmad et al., 2016; Kaikabo et al., 2017). There is also a notable amount of research on *in vitro* testing of phages, including the testing for the phage’s host range, biocontrol assays against pathogens, and phage formulation techniques. We retrieved a few publications on *in vivo* testing of phages using invertebrates, such as moth (Wintachai et al., 2019; Nale et al., 2021) and oysters (Le et al., 2020); *in vivo* vertebrate model systems included zebrafish (Assafiri et al., 2021), catfish (Le et al., 2018; Dang et al., 2021), tilapia (Dela Cruz-Papa et al., 2014, 2017), chicken (Wong et al., 2014; Kaikabo et al., 2017), and mice (Guang-Han et al., 2016). Lastly, a few publications focused on the applications of phages in combating MDR organisms. For instance, SEA researchers discovered and characterized phages with activity against MRSA (Dakheel et al., 2019; Tan et al., 2020), ESBL-producing *E. coli* (Adamu Ahmad et al., 2016), MDR *K. pneumoniae* (Cornista et al., 2019; Assafiri et al., 2021), MDR *A. baumannii* (Wintachai et al., 2019; Styles et al., 2020), MDR *Salmonella* species (Pelyuntha et al., 2021), and *Clostridioides difficile* (Phothichaisri et al., 2018). The efficacy of these phages has yet to be confirmed clinically. Currently, global case reports lean towards phage therapy applications for bone, surgical, and other local infections, with several cases also reporting usage in critical and systemic infections, especially those caused by MDR organisms. With the region’s current strength in basic and genetic characterization of phages and growing expertise in *in vitro* and *in vivo* testing of localized external infections in animals, we expect that the future clinical applications of phage therapy in the region would also lean towards the treatment of surgical and skin infections of known etiology. Unfortunately, at the time of writing our review, the region had no published case report on any phage therapy applications in humans.

## Considerations in Phage Therapy

Not all phages are ideal for therapeutic applications. Therapeutic phages should be obligately lytic to ensure killing of the target pathogen (Harper and Enright, 2011; Wittebole et al., 2014; Lehman et al., 2019). The phage should have a high rate of adsorption to the target pathogen, a short generation time (Drulis-Kawa et al., 2015), and species-specific activity (Khawaja et al., 2016). As in the case with typical antimicrobial agents, scientists also recommend profiling the phages in a phagogram or a phage equivalent of an antibiogram (Barbu et al., 2016). A phagogram refers to the continuous testing of the phage efficacy against a defined collection of pathogens called a pathogen library or diversity panel (Deresinski, 2009; Casey et al., 2018). Phagogram profiling will ensure that the therapeutic phages are specific to their target pathogen. Standardized phagogram profiling is not yet incorporated in most *in vitro* and *in vivo* testing of phages discovered in SEA, although specificity testing in multiple strains of target pathogens have been done (Abdulamir et al., 2015; Phothichaisri et al., 2018; Dakheel et al., 2019; Tan et al., 2020).

Therapeutic phages are usually combined with storage media to produce a phage formulation. As with other therapeutics, phage formulations should be prepared according to current good manufacturing practices (GMP) and established quality assurance standards. Phage formulations should neither have impurities nor contaminants (i.e., endotoxins and host cell proteins; Jennes et al., 2017; Furfaro et al., 2018; Lehman et al., 2019). Since the delivery route influences the efficacy of phage therapy, various studies have explored different ways of phage administration for therapeutic purposes. One common way to administer phage therapy is through topical applications for skin and burn infections (Morozova et al., 2018). Oral phage administration is also a common scheme (Kakasis and Panitsa, 2019). Considering that the phages will be taken internally, they should be stable at human body temperature (35–39°C) and physiological pH (4–9; Jin et al., 2012). Other factors, such as stability in low gastric pH and bile salts, should also be considered (Letkiewicz et al., 2010). Hence, phage formulations can include bicarbonate water or be enclosed in a gel (Kakasis and Panitsa, 2019). In a study in Malaysia, researchers constructed chitosan nanoparticles loaded with phage ΦKAZ14 as an alternative oral delivery platform in chickens. They indicated that the chitosan-phage nanoparticle is more stable, *in vivo*, compared to standalone phage (Adamu Ahmad et al., 2016). An extension of this study showed that the same nanoparticle controlled the bacterial colonization of avian pathogenic *E. coli* in chicken and decreased the symptoms of colibacillosis (Kaikabo et al., 2017). The applicability of using this nanoparticle-based oral delivery method in clinical applications can be explored in SEA since the technology and knowledge for creating it is already being explored in Malaysia. Other administration routes for therapeutic phages include the respiratory route through intranasal spray (Cao et al., 2015; Casey et al., 2018), systemic route through intraperitoneal or intramuscular injection (Criscuolo et al., 2017; Jennes et al., 2017), or rectal route through fecal microbiota transplant (Letkiewicz et al., 2010; Broecker et al., 2013). These administration routes are yet to be explored in SEA, *in vivo* or clinically.

Similar to antibiotic therapies, the pharmacodynamic properties of phages must be determined to establish an effective dosing regimen for the successful treatment of severe infections. Proper phage dosage ensures that an appropriate amount of the virus will encounter the target pathogen in the infection site (Zhang et al., 2018). In an inundative therapy (passive treatment), a high titer (10^8^–10^10^) of phages is administered to the patients (usually in single-dose regimen) to kill a large number of the target pathogen (Danis-Wlodarczyk et al., 2021). Although some studies aligned with this strategy (Zhang et al., 2018), opposing views caution that a too high number of phages may result in “lysis from without,” or the non-specific lysis of cells caused by phages (Barbu et al., 2016; Cieplak et al., 2018). More studies are needed to confirm this phenomenon. Meanwhile, an active therapy depends on the *in situ* amplification of phages to achieve the inundative concentration for bacterial clearance (Danis-Wlodarczyk et al., 2021). No formal study on phage pharmacodynamics has been done in the SEA population.

Biofilm formation is an important virulence factor in most MDR pathogens. Remarkably, phages have been studied for their activity against bacterial biofilms. *In vitro* studies in SEA showed that certain bacteriophages degrade biofilms of *A. baumannii* (Wintachai et al., 2019), *E. coli* (Jassim et al., 2012), *K. pneumoniae* (Wintachai et al., 2020), and *S. aureus* (Abdulamir et al., 2015; Dakheel et al., 2019). This activity can be attributed to the production of various hydrolases (i.e., extracellular polymeric substance depolymerases) in the capsid that can effectively disrupt and disperse bacterial biofilms (Lin et al., 2017). Some phages would also have tail fibers with inherent depolymerase activities (Dufour et al., 2016). A deeper understanding of the role and mechanisms of phages in disrupting biofilms remains to be gained, as *in vivo* studies and reports in the clinical setting have yet to be described.

Finally, phage resistance is another aspect that should be considered in phage therapy. Researchers believe that the evolutionary pressure exerted by the phage greatly exceeds any resistance mechanisms acquired by the target bacteria (El-Shibiny and El-Sahhar, 2017; Harper, 2018). In addition, the rapid evolution of phages helps improve their virulence towards the target bacteria, making adaptive bacterial resistance ineffective to phages (Golkar et al., 2014). However, the occurrence of phage-resistant bacteria is inevitable and should be considered when designing phage therapy treatment (Cairns and Payne, 2008; Casey et al., 2018). For instance, a *P. aeruginosa* small colony variant resistant to phage PB1 has been described in Singapore (Lim et al., 2016), but its susceptibility to other phages has not been explored. To prevent phage resistance, one should be on the lookout for bacterial mechanisms against phages after administering phage therapy. These mechanisms may include the CRISPR-Cas system, superinfection exclusion, and abortive infection. Phage cocktails could also be explored as an option to help prevent the rise of phage resistance, as we will describe later. In summary, the considerations in phage therapy should be observed starting from the characterization of the isolated phages, their preparation, and the administration to the patients (Supplementary Figure 3).

## Perspectives of Phage Therapy in SEA

Frederick Twort and Félix d’Hérelle independently observed the bactericidal activity of phages in 1915 and 1917, respectively. In 1919, d’Hérelle used phages to treat bacterial infections, marking the start of non-randomized trials worldwide and launching the term “phage therapy” (Wittebole et al., 2014; Criscuolo et al., 2017). In SEA, the earliest documented phage therapy application was from the expeditions of d’Herelle and colleagues in Laos and Vietnam from 1916 to 1930 (Chanishvili, 2012). According to the historical accounts, d’Herelle used phages isolated from plague-infected rats to treat plague victims and introduced phages from cholera patients into village wells to decrease mortality rates from cholera in the region (Vandamme and Mortelmans, 2019).

Despite the decline in the medical field’s enthusiasm on phage therapy (i.e., due to the discovery of penicillin and lack of experimental support for phage) in the late 1940s, phage therapy centers were continuously built in the USSR, Georgia (George Eliava Institute, 1916), Poland (Hirtszfeld Institute; Institute of Immunology and Experimental Therapy of Polish Academy of Sciences, 1952), and France (Pasteur Institute in Lyon and Paris, 1917). In SEA, the Phage Directory (https://www.phage.directory/, accessed on 20 December 2021), an online directory to find and track phage laboratories worldwide, conceptualized and maintained by an independent, two-person organization, recorded several phage laboratories and phage collections, but no registered phage therapy center. The listed phage laboratories are found in Indonesia (University of Jember-KeRis Terapi Bakteriofag), the Philippines (University of Santo Tomas-Papa Lab), and Thailand (Silpakorn University-Akoy Lab, Kasetsart University-Kasetsart Phages). In Malaysia, the Center for Excellence for Omics-Driven Computational Biodiscovery (COMBio) of the Asian Institute of Medicine, Science and Technology also explores phages isolated from rainforests against key human pathogens (Rajandas et al., 2021). Meanwhile, the listed phage collections are found in Indonesia (University of Jember-KeRis Terapi Bakteriofag Phage Collection) and Thailand (Silpakorn University-Nasanit/Akoy Lab Phage Collection) only. Some institutions and phage laboratories not listed in the Phage Directory, but whose members contributed to recent biomedical and pre-clinical research literature on phages, can be found in Indonesia (Atmajaya Catholic University, Brawijaya University, Universitas Airlangga), Malaysia (International Medical University, Monash University Malaysia, Universiti Putra Malaysia, Universiti Sains Malaysia, University of Malaya), Philippines (University of the Philippines), Singapore (Nanyang Technological University, National University Singapore), Thailand (Chulabhorn Research Institute, Chulabhorn Royal Academy, Chulalongkorn University, Mae Fah Luang University, Mahidol University, Prince of Songkla University, Srinakharinwirot University, University of Phayao, Walailak University), and Vietnam (Ho Chi Minh City University of Technology, Vietnam National University). Although the list of institutions and laboratories presented in this review is based only on what is retrievable in PubMed (https://www.pubmed.ncbi.nlm.nih.gov/, accessed 20 December 2021) and may not be complete, this report shows that there is an interest among SEA academic laboratories in studying phages.

In 2009, the Nestlé Research Center (Switzerland) started the first randomized, double-blind, placebo-controlled phase I trial on the safety of phage therapy in Bangladesh. The study reported that orally administered phages do not affect the normal flora of the participants, particularly the native *E. coli* populations (Sarker et al., 2016). In SEA, a clinical trial in Singapore evaluated phage therapy in wound care for critical limb ischemia. This trial, registered as “ongoing” at the clinical trial registry of the Singapore Health Sciences Authority (HSA, https://www.eservice.hsa.gov.sg, accessed 20 December 2021) in 2015, was sponsored by D&D Pharma (Pte.) Ltd. (Protocol number SGH001). The Singapore General Hospital and Mount Elizabeth Novena Hospital & Specialist Centre served as the trial sites. However, the trial’s interim data or publications were unavailable when writing this review. There is currently no registered clinical trial that explicitly targets multidrug-resistant pathogens in the region (based on www.clinicaltrials.gov as of 20 December 2021).

Although clinical studies on phage therapy are lacking in SEA, *in vivo* pre-clinical studies have been reported. For instance, phages have been shown to successfully control *A. baumannii* (Wintachai et al., 2019) and *Salmonella* spp. (Nale et al., 2021) in *Galleria mellonella* larvae. These findings are initial evidence of phage efficacy in reducing surface colonization of pathogens in animals, including humans. Researchers in SEA also showed the safety of using phages in controlling *K. pneumoniae* infection in *Danio rerio* larvae (Assafiri et al., 2021). Another study of lung infections caused by *B. pseudomallei* in mice indicated that phages administered peritoneally successfully protected the animals from disease progression and mortality (Guang-Han et al., 2016). Other *in vivo* phage therapy studies focused on treating infections in poultry (Wong et al., 2014; Kaikabo et al., 2017) and aquaculture (Dela Cruz-Papa et al., 2014, 2017; Le et al., 2018; Dang et al., 2021). The implications for clinical use, however, may not be apparent especially if the bacterial pathogen is specific only to the animal (i.e., *A. hydrophila* in freshwater fishes, avian pathogenic *E. coli* in birds, and *Vibrio alginolyticus* in oysters). However, with the potential zoonotic transmission of infections, the expanded use of specific phages for treating both animals and humans could be possible.

The lack of actual clinical experience, established phage collections, and phage consortium in SEA are gaps of knowledge that need to be filled, thus providing an opportunity to build the foundation for shifting the paradigm of treatment. In the next section, we propose systematic strategies to start a phage revolution in SEA and invite more biologists, biomedical scientists, and clinical decision-makers in the region to explore phage therapy against MDR organisms.

## Strategies for Phage Revolution in SEA

**Figure 2:**
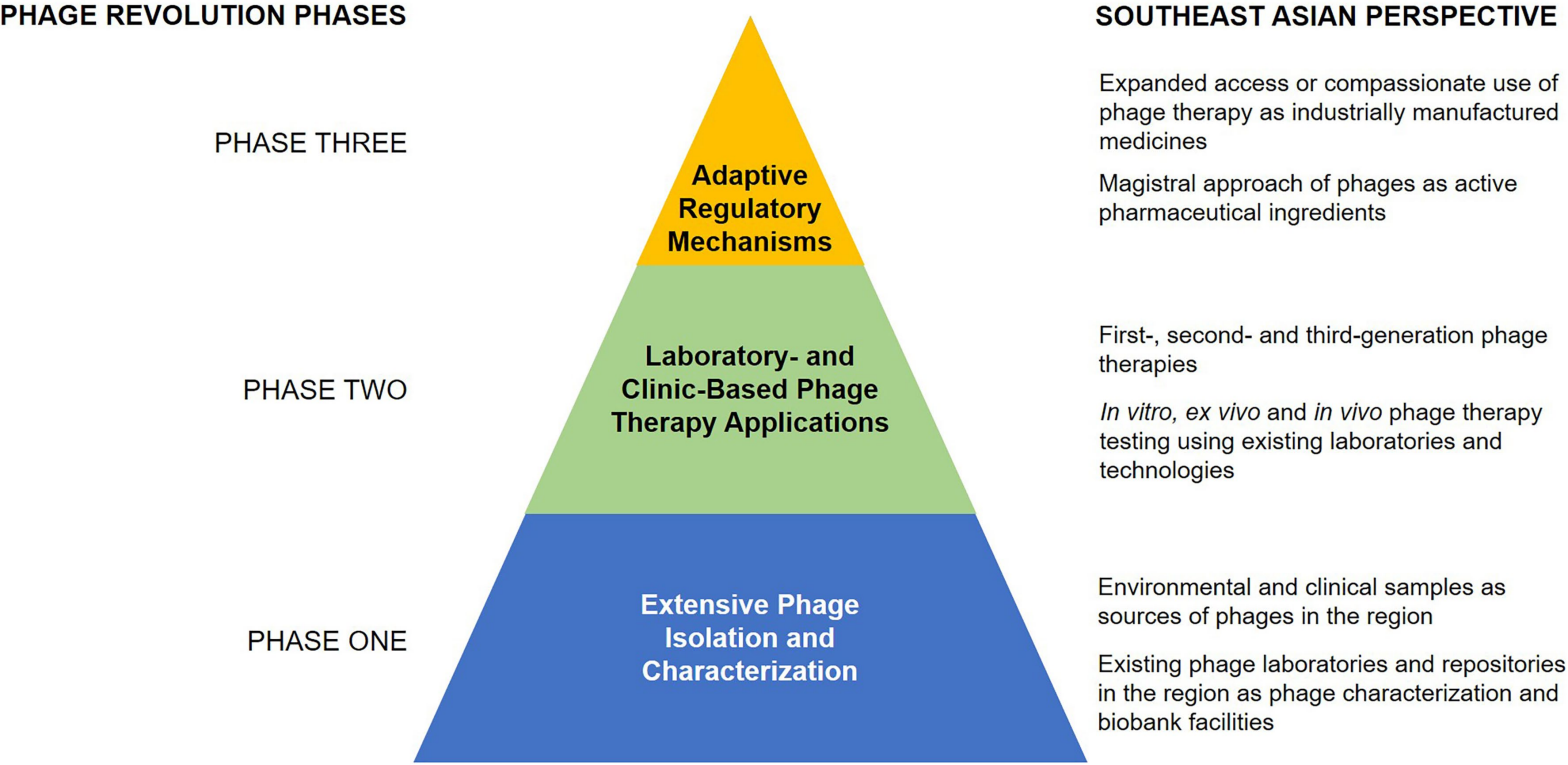
A three-phase strategy for implementing a phage revolution, with perspectives for SEA.

A structured research and implementation approach is necessary to encourage phage therapy against multidrug-resistant infections in SEA (Figure 2). The first phase involves the extensive isolation, characterization, and matching of phages to their target pathogen, guided by the existing knowledge on phage biology and the mechanisms of phage infection. The second phase involves designing appropriate models, both laboratory-based and clinical-based, to implement phage therapy protocols. Lastly, establishing regulatory guidelines for standardized phage therapy will be the peak phase of the revolution, which could hopefully pave the way to create appropriate regulations for phage therapy in SEA.

### Phase One: Extensive Phage Isolation and Characterization

Starting the phage revolution entails mastery of phage isolation and characterization. As discussed earlier, isolation and characterization of phages involve determining their morphology, lytic activity, specificity, generation time, and adsorption rates using traditionally accepted methods. These methods remain to be useful but present certain limitations. We summarized these limitations in Supplementary Table 1, but a thorough discussion on this topic is presented by Hyman (2019). With the advancements in instrumentation and understanding phage biology, other non-traditional methods can be explored to improve phage isolation and characterization further. For instance, specific samples could be explored in isolating phages with targeted activities. As discussed earlier, phages can be isolated practically in all types of samples. However, to acquire phages with activities against MDR clinical pathogens, we propose exploring the sites heavily contaminated with clinical samples and hospital sewage, as indicated in some studies (Kakasis and Panitsa, 2019). Hospital wastewater systems are the most readily accessible sites for this purpose. Meanwhile, clinical samples, such as blood, stool, and urine, can also be used as the primary source of phages (Letkiewicz et al., 2010; García-Quintanilla et al., 2013). We believe that this approach is more straightforward in isolating specific phages, given that the clinical samples contain the actual bacterial target. Working on the ecological principle that predators are present where the prey is, phages can also be isolated from infectious samples (Chibani-Chennoufi et al., 2004). Despite this proposal, we do not discourage exploring other potential sources of phages for clinical applications. As proposed by Rajandas et al. (2021), other biodiverse habitats, such as the rainforests, which are commonly found in SEA, can be explored as sources of clinically relevant phages. This notion is supported by the isolation of phages with activities against clinical pathogens from farms (Wongsuntornpoj et al., 2014; Sellvam et al., 2018), freshwater (Al-Fendi et al., 2014; Wangkahad et al., 2015), marine environment (Guang-Han et al., 2016), and soil (Shan et al., 2014; Withatanung et al., 2016) in SEA. There is now also increasing availability of widely studied phages with known activities against clinical infections. These phages are curated or produced by several phage organizations as listed in Phage Directory (https://phage.directory/orgs, accessed on 20 December 2021) and The Bacteriophage Ecology Group (http://companies.phage.org/, accessed on 20 December 2021). To promote access to these phages, a mechanism to collaborate with these external phage resources should be established in SEA.

With the advancement in molecular biology, additional phage characterization steps before therapeutic use are now possible. Some scientists suggest structural protein analysis for each phage using sodium dodecyl sulfate polyacrylamide gel electrophoresis (SDS-PAGE), although without suggested practical reason (Kwiatek et al., 2015). Phage protein profiling can aid in phage classification, bioengineering, and identifying potential immune reactions; however, more studies are needed to establish these applications. With the movement to pursue genome-based phage classification (Turner et al., 2021), we would also recommend whole-genome sequencing (WGS) for the characterization of newly discovered phages. WGS can be used as a crude screening method for choosing the ideal therapeutic strain. In SEA, novel phages have been screened using WGS for their lysogenic and endotoxin production potentials (Gan et al., 2013; Lal et al., 2016a; Wirjon et al., 2016; Thanh et al., 2020; Dewanggana et al., 2021; Nuidate et al., 2021), which are important properties to avoid in choosing therapeutic phages. WGS can also aid in the more detailed classification and identification of the phages, as also applied in SEA strains with promising therapeutic potentials (Gan et al., 2013; Hoai et al., 2016; Lal et al., 2016a,b; Wirjon et al., 2016; Sellvam et al., 2018; Handoko et al., 2019; Thanh et al., 2020; Tu et al., 2020; Nuidate et al., 2021). Metagenomic sequencing of phages in the prospect samples will be helpful in the initial screening of phage genomes even before the isolation of the actual virions. This strategy has been used in studying phages found in Brunei and Malaysia (Kerfahi et al., 2019).

In terms of preparing the phage formulations, extensive optimization and characterization are also warranted. For phage delivery platforms, different methods can be studied to optimize phage stability and activity. For instance, researchers in Thailand used novel microencapsulation of phage cocktails against *S. typhimurium via* freeze-drying (Petsong et al., 2021). The researchers also showed that microencapsulated phages are more stable and elicit similar efficacy against the target bacteria in an *in vitro* surface contamination experiment (Petsong et al., 2019). To deliver phages *via* systemic routes, one should consider phage clearance by the immune system. In an *in vivo* study in Malaysia, an intraperitoneal phage administration was found to be effective in reducing *B. pseudomallei* infection in mice (Guang-Han et al., 2016). Although the formulation appears tolerable among mice, the appropriate concentration to be used and the immune interaction has yet to be formally described, more so if administered to humans. Scientists recommended optimizing the phage titer in preparations for every strain and target (Górski et al., 2015). However, the kinetics of phages in human peripheral blood is still not established (Schooley et al., 2017). We believe it will be beneficial to consider a personalized pharmacokinetics study of the specific phages for systemic infections. As different patients may have different rates of phage clearance, trials of multiple phage dosages may be necessary (Lin et al., 2017). As mentioned earlier, an appropriate phagogram profiling should also be conducted for each new phage. The phagogram profiling should be done on all potential strains of the target pathogens.

In addition to understanding phage formulation components and qualities, phage banks should also be established in SEA. Although university-based phage collections are present in the region as described earlier, the phage revolution could benefit from maintained and monitored phage repositories that could act as the central source of viruses for therapy. Like any other biobank, the creation and maintenance of phage banks require equipment and process investments. Although they are not widespread, ultralow-temperature freezers (−80°C) are already present in SEA and could serve as a starting point for the long-term storage of promising specimens. Otherwise, efficient preservation methods, such as the use of pH-neutral solutions with low ionic contents, or lyophilization with sugar stabilizers can also be explored for long-term storage with less equipment requirements (Duyvejonck et al., 2021). The quality assurance systems and protocols also need further attention; these are currently non-existent for the biobanking of phages in the region. For instance, the proper delineation and characterization of the banked phage (master seed lot), phage products (working seed lots), and therapy-ready preparations need to be considered (Pirnay et al., 2015; Pelfrene et al., 2016).

### Phase Two: Laboratory- and Clinic-Based Phage Therapy Applications

The second revolutionary phase entails the appropriate modeling and implementation of phage therapy. Modern applications of phage therapy not only include the traditional use of phage formulations but also the use of combination therapies, phage products, and other phage-related interventions. Krylov and colleagues differentiate these modern applications into three generations of phage therapy products (Krylov et al., 2015). In our current review, we enriched the previous definitions by referring to the first-generation phage therapy as the traditional phage-based therapy, the second-generation as the use of phage-derived biologics and combination therapy, and the third-generation as the use of bioengineered phages. We summarized these applications in Table 3.

**Table 3:** Phage therapy products and its applicability in SEA.

Phage therapy products	Characteristics	Limitations	Applicability in SEA
First generation	Phages isolated from environmental enrichment Clinical relevance: most basic of phage therapy applications. Monotherapy or phage cocktail regimes in simple infections or as compliment to existing antimicrobials	In-depth characterization of phages before using in actual therapies High-throughput screening requirement	Most applicable in SEA and other regions initially starting to utilize phage therapy
Second generation^1^	Phage products (i.e., enzymes) with potential antibiotic properties OR combination of first-generation phages with other antimicrobials Clinical relevance: more advanced form of phage therapy application. Involves protein purification and additional activity optimization. Phage therapy for simple or complex infections	Purification of phage proteins and additional testing requirements Testing of phage and antimicrobial combinations for compatibility and synergistic activities	Applicable to SEA once advanced manufacturing and quality testing has been established
Third generation	Bioengineered phages (partially or entirely) Clinical relevance: most advanced form of phage therapy applications. Relies on bioengineering and more rigorous testing. Personalized phage therapy or therapy for specific infections with no known therapy	Requirement for genomic characterization of the phages and identification of genes that may affect antimicrobial activity Lack of expertise in genetic modification of phages High turnaround testing requirement of designed phages	Applicable in SEA once advanced knowledge and expertise on phage genetics and recombineering has been established

1 The original paper of Krylov et al. (2015) described second-generation phage therapy products as mixtures of previously characterized lytic phages or combination of lytic and pseudo-temperate phages. As it may have no clear distinction with the first generation in terms of the actual product to be used in therapies, we proposed a new definition for this generation to refer to the current characteristics described in the table.

The first-generation phage therapy is the most studied. Traditionally, phage therapy can be done using a monoculture of a single specific phage against uncomplicated infections. However, as scientists began to address complex infections, they considered the use of phage cocktails. Phage cocktails refer to the combination of various phage strains or clones in a single preparation (Burrowes et al., 2011). In SEA, cocktails have been used for *in vitro* and *in vivo* therapeutic applications. For instance, Phothaworn et al. (2020) used a two-component phage cocktail (SE-W109 and ST-W77) to increase the efficacy and breadth of *Salmonella* serovars killed by the formulation. Similarly, Dela Cruz-Papa et al. (2017) used a two-component phage cocktail (UP87, B614) from the Philippines to increase the efficacy of *A. hydrophila* clearance in *Oreochromis niloticus* compared to monotherapy. These properties could be beneficial in treating complex infections caused by different bacterial strains. Others indicate that phage cocktails may help prevent or combat phage resistance among the targets. In a recent study by Nale et al. (2021), a three-component cocktail (with the same two phages as used previously by Phothaworn et al., 2020) exhibited effective clearance of phage-resistant *Salmonella*, *in vitro*, possibly due to complementary activities of the phages in the formulation. This property could further be explored in justifying the utility of phages for long-term therapeutic applications without worrying about phage resistance. Banking on the high specificity of phage cocktails, it can be customized based on the pathogen profile of the infection from patient sample cultures (Lin et al., 2017; Furfaro et al., 2018). Logistically, phage cocktail preparations may be more costly and time-consuming since the characteristics (i.e., life cycle) and properties (i.e., stability in preparations and in the human body) of the phage must be matched and complementary with each other (Cairns and Payne, 2008; Krylov et al., 2015; Lin et al., 2017). This characterization is vital to ensure the component phages’ orchestrated modes of action (Cairns and Payne, 2008).

Second-generation phage therapy involves the use of phage-derived products, such as lytic enzymes. Phages use lytic enzymes to hydrolyze the cell wall of their host bacteria, allowing for the release of viral progenies. The enzymes, including holins and endolysins, are possible phage-based pharmaceuticals in the future (Lin et al., 2017). In particular, these proteins are seen as potential antimicrobials because of their low-dosage potency and high target specificity similar to phages. Mass production of lytic enzymes is also easier using traditional recombinant techniques (Lin et al., 2017). In recent years, engineered lytic enzymes have been explored to induce highly specific or broad-spectrum cell wall cleavage. In Singapore, chimeric lysins composed of different segments from LysEF-P10 (targets *Enterococcus faecalis*) and PlyV12 (targets *Streptococcus* sp., *Staphylococcus* sp., and *Enterococcus* sp.) have been successfully engineered. The researchers showed that the engineered lysin called P10N-V12C (with cell wall-binding domain of PlyV12) have both a broader lytic activity against enterococci, streptococci, and staphylococci, and a specific activity against *E. faecalis*, *in vitro* (Binte Muhammad Jai et al., 2020). In the future, the actual application of lytic enzymes to treat infection warrants additional *in vitro* and *in vivo* studies.

Some researchers believe that antibiotic and phage activities do not interfere with each other because of their different modes of action. Antibiotic resistance may not affect phage therapy efficacy (Zhang et al., 2018). One hypothesis is that a more substantial antimicrobial effect can be achieved if clinicians combine antibiotics and phages into one therapeutic regimen (Viertel et al., 2014; Torres-Barceló, 2018). First observed in 2007, researchers reported that a minimal dose of an antibiotic could stimulate the therapeutic activity of phage in *E. coli*, a phenomenon described as the Phage-Antibiotic Synergy (PAS; Comeau et al., 2007). Scientists in Indonesia investigated PAS in reducing the load of *E. coli* and *Salmonella* spp., *in vitro*. Their results showed that phage фPT1b combined with amoxicillin and tetracycline at specific ratios successfully increased the lysis of different *E. coli* strains. These findings indicate a reduced level of resistance to the antibiotics (Narulita et al., 2020). Similar observations have been reported with the combination of phages ϕSZIP1, ϕSZIP2, and ϕSZUT plus cefadroxil against *Salmonella* sp. *Escherichia* sp. and *S. aureus* (Iqbal et al., 2020) and with phage P22 plus ciprofloxacin against *S. typhimurium* (Petsong et al., 2018). Scientists hypothesized that the initial antibiotic dose elongates the bacteria, making it easier for phages to bind to them due to the increased surface area of the pathogen (Torres-Barceló, 2018). Using this “combination therapy,” the lifespan of exhausted, early generation antibiotics can be prolonged since only its initial effects on the pathogens (not necessarily the bactericidal activity) are needed to achieve the therapeutic effect desired (Iqbal et al., 2020; Narulita et al., 2020). In addition, it could help increase the activity of the antibiotics against polymicrobial interactions like biofilms, although no investigations in SEA have been made to confirm this activity. The actual clinical performance of combined therapy using antibiotics and phages has yet to be investigated in SEA.

The genetic characteristics of phages make them ideal for bioengineering, eventually giving rise to third-generation phage therapies. The phage display technique involves fusing antibody variants or peptides to proteins of the phage coat proteins; iterative rounds of screening then enhance the phage coat’s affinity for target (Criscuolo et al., 2017). DotBio, a start-up biotechnology company in Singapore, utilizes the phage display technique to produce DotBody technology proposed to be used in multi-functional therapies (https://www.dotbio.com/en/technology, accessed 20 December 2021). Meanwhile, scientists from SEA reported bioengineered phages with applications as vectors for selective delivery of transgenes in mammalian cells *via* phage surface modification (Namdee et al., 2018; Chongchai et al., 2021) and dual tumor targeting by hybridization of an adeno-associated virus with the phage capsid (Przystal et al., 2019). However, no specific modifications have been done to create phages specifically targeting clinically relevant bacteria. Hence, although scientists in SEA engineer phages for clinical applications, they still have to explore engineered phages in combating MDR organisms. Among the avenues that can be explored in the region is the genetic programming of phages to deliver CRISPR/Cas system to the target multidrug-resistant bacteria. This technique can be useful in disrupting the expression of antibiotic resistance genes of the host, making them more susceptible to antibiotics, and preventing the spread of antibiotic resistance genes (Lin et al., 2017). Aside from CRISPR/Cas, phages can also be engineered to kill the host by delivering other lethal genes encoding for lytic enzymes, such as endonucleases, lytic enzymes, and toxins (Viertel et al., 2014). Finally, a whole phage particle can also be constructed, eliminating the need to isolate and purify bacteriophages. These “designer phages” can be used to incorporate all positive traits of bacteriophage for therapeutic use. Constructing designer phages is still an uncharted niche in SEA.

To address the need for rigorous testing of phages before clinical use, extensive laboratory testing for safety and efficacy is necessary (Nale and Clokie, 2021). Laboratory testing of phages should be conducted at three levels – *in vitro*, *ex vivo*, and *in vivo* (Supplementary Figure 4). For *in vitro* testing, the characterization of phages and optimization of phage formulations should be given priority as described in the previous section of this review. This level of testing should also include the production of phage formulations that will be used in the succeeding testing stages. We believe that the first level of testing phages will not be a problem in SEA since the academic institutions and existing phage laboratories regularly conduct phage testing and characterization. However, we propose to develop standard laboratory protocols for the use of different phage laboratories to ensure that the manufacturing process can be standardized, monitored, and regulated in the future. These protocols should also follow the current GMP principles (Moelling et al., 2018). The second and third levels of testing will be more challenging since it involves using live cell cultures, tissue models, and live animals. Researchers recently used human cell lines in studying the dynamics and interaction of phages and mammalian cells infected with the target bacteria (Møller-Olsen et al., 2018; Shan et al., 2018). However, a more sophisticated method of testing the efficacy of phages is to mimic the human organ environment (*ex vivo*) affected by the bacteria by using infection models. A recent study reported this approach to infer that an enterococci phage cocktail is effective in a collagen wound infection model (Melo et al., 2019). Another *ex vivo* approach used controlled and maintained batch fermentation setups to simulate the complex internal environment of the animals (or humans) when studying phages to be administered internally (Rivas et al., 2010; Nale and Clokie, 2021). *Ex vivo* approaches in studying phage efficacy and safety have yet to be done in SEA. The use of animal models (*in vivo*) in testing phage efficacy remains an accepted method in the scientific community. Animal models ensure that proper phage delivery methods in living systems and immune response dynamics will be considered when phage therapy is administered (Nale and Clokie, 2021). The choice of animal is critical in ensuring that the human infection pathogenesis and environment can be mimicked accurately. As discussed earlier, *in vivo* phage therapy studies have been conducted in SEA, but the transition from doing pre-clinical to clinical research in phages has yet to be done.

The past five years saw the advent of three-dimensional cellular constructs mirroring the functions of human tissues and organs (a general term called tissue engineering and regenerative medicine or TERM) in SEA, primarily by groups in Singapore (Han et al., 2020). With TERM, the option of using bioprinted infection models for phage therapy arises. Bioprinting refers to fabricating three-dimensional tissue constructs using biomaterials and living cells (Gungor-Ozkerim et al., 2018). We believe that exploring this option would be beneficial in a personalized therapeutic approach by providing an avenue for laboratories to test the dynamics and efficacy of the phage formulations in patient-derived cells bioprinted to form tissues or organoids and infected with a clinical isolate from the same patient. Using this approach, researchers may reduce the amount of animal testing required, while the *ex vivo* environment may reflect the actual infection environment in humans more accurately than in animal models. In addition, patient cells and patient-derived bacterial pathogens will be used for testing. We believe that this approach is also complementary to the refined *in vivo* approach (i.e., use of invertebrate models), as suggested in other studies (Nale et al., 2015, 2021; Brix et al., 2020).

### Phase Three: Adaptive Regulatory Mechanisms

The third phase of the phage revolution involves creating standards and guidelines for the therapeutic use of phages. Currently, regulatory agencies in most parts of the world, including SEA, have not officially approved any phage-based products for use in humans. Experts attributed this situation to the fact that no standardized definitions, regulations, and guidelines are available for phage formulations (Wittebole et al., 2014; Krylov et al., 2015; Morozova et al., 2018). In addition, other regulatory standards in the SEA and gaps for implementation have been observed, potentially affecting the progress of phage-based therapeutics as an accepted therapeutic option (Table 4). Although combating antimicrobial resistance is among the top priorities of the ASEAN health cluster for 2016–2020, phage therapy is not among the strategies listed (Association of Southeast Asian Nation, 2021). Given that there are no formal regulatory discussions on phage therapy applications, we hope that this paper could start a dialogue between the different stakeholders in the region. The initial movement towards this goal has been started by the implementation of the inaugural symposium on phage and phage-derived technologies in Singapore last December 2021, organized by Cellexus. Other activities, such as the first Virtual SEA Phage Workshop to be administered by Phages for Global Health in 2022 and the Protein Engineering and Phage Display Conference to be launched in Malaysia in 2023, are also on the way. We are optimistic that additional regional activities towards phage use and appreciation can be done in the next few years once discussions continue to progress.

**Table 4:** Existing regulatory standards with potential implications in phage therapy implementation in SEA.

ASEAN regulatory standard^1^	Gaps in implementation^1^	Implications in phage therapy regulation
Medicines registration is required to obtain pharmaceutical product marketing license	Lack of technical expertise, institutional capacities, and long timelines for approval	Non-consensus on the definition of phages either as industrially manufactured medicine or as an active product ingredient makes the current regulatory guidelines non-applicable or not very clear when applied to phage therapy
Regional or local registration is required for innovative medicines newly introduced to the global community	Drug lag of more than 3 years due to additional regulatory burden to manufacturers and lack of technical expertise in the ASEAN for innovative medicines	Lack of technical expertise in phage therapy as an innovative medicine potentially resulting in longer lag in regulatory approval
Expanded access or compassionate use cases allowing the administration of unregistered products to select and special patients with no other treatment option or ineligible to clinical trials	Expanded access or compassionate use of drugs as a circumventing mechanism to the regular medicine registration pathway	Non-sustainable option for using phage therapy potentially delaying the appreciation of the public to the therapy. Decrease interest of industries in high-throughput manufacturing and production of phage formulations
Product development needs to follow harmonized and streamlined standards and guidelines (i.e., process validation, stability testing, and bioavailability/bioequivalence)	Shortage in resources and expertise for implementation, and differences in existing local pharmaceutical laws and regulations	Lack of technical expertise and resources in phage therapy potentially hindering early efforts in streamlining of standards and guidelines for phage formulation development
Establish medicines regulatory harmonization agenda	Fragmented approach in implementation of the harmonization initiatives and lack of political engagement hindering the progress of the agenda	Lack of standardized basic definitions and protocols for phage therapy hindering the establishment of a regulatory agenda for phage therapy

1 Based on the report of Teo et al. (2016) on medicines regulatory systems and scope for regulatory harmonization in Southeast Asia.

Despite the current situation in SEA, global regulatory agencies like the US-Food and Drug Administration (US-FDA) and the European Medicines Agency (EMA) provide avenues for the compassionate use of phages as expanded access investigational (i.e., unregulated) new medicines. In these cases, phage formulations can be administered to patients on a per case basis, but the actual scheme of facilitating compassionate phage therapy remains highly variable (McCallin et al., 2019). For patients with financial resources and capacity to travel, phage therapy can also be accessed by traveling to countries where phage therapy is an approved therapeutic option (i.e., Georgia). It is important to note that the regulatory agencies in these regions consider phages as industrially manufactured medicines. Therefore, phages can be treated like traditional drugs, which should follow GMP standards for manufacturing, undergo efficacy testing in clinical trials, and obtain marketing clearance (Brives and Pourraz, 2020). In ASEAN countries, expanded access or compassionate use of unregistered treatment options, including phage therapy, has also been a viable option (Teo et al., 2016). ASEAN countries reported high rates of compassionate use of unregistered medicines, but the use of phage therapy even in critical cases has not been reported. Although compassionate use exists as a safety net, we believe that this workaround is not a sustainable regulatory solution in the long run because: (1) it limits the use of phage therapy for special or emergency cases only, and (2) it creates an impression that phage therapy is only a last-line treatment option, preventing progression in terms of acceptability to the pharmaceutical industries and the general public.

Pirnay et al. (2018) described a magistral approach as a model of regulating phage therapy, where phages are considered active product ingredients instead of industrially manufactured medicines. This approach, currently applied in Belgium, involves the collaboration of phage formulation manufacturers (can be individual hospital pharmacies) and Belgian-Approved Laboratories in ensuring the production of high-quality phages for human use. In this approach, individual hospitals can access phages from biobanks that underwent quality control measures by the Belgian-Approved Laboratories (Moelling et al., 2018). The manufacturers have leeway on the actual production of the phage formulation, as long as it follows the standards of current GMP or the like. This attribute opens the possibility of personalized phage formulations for individual patient management. The Belgian-Approved Laboratories serve as the quality control body that assesses and certifies the constitution of phages before they can be given to the manufacturers for magistral preparations. In SEA, the magistral approach can be considered as soon as the following requirements are satisfied: (1) establishment of well-maintained and monitored phage banks (which will be the source of phages or active pharmaceutical ingredient), (2) appointment of “ASEAN-Approved Laboratories,” and (3) training of pharmacies and independent manufacturers in producing magistral preparations of phages following GMP. For the first requirement, existing phage banks in academic institutions could be tapped, or a central phage biobank could be established, accessible to SEA countries. For the second requirement, each ASEAN member could delegate existing public agencies or private institutions with capabilities to conduct quality control testing for phages. Virology institutes with access to advanced phage characterization equipment like electron microscopes and sequencing platforms would be ideal for this purpose. For the last requirement, hospitals and interested manufacturers should be trained and certified for GMP standards and phage formulation protocols. A consortium of experts in the SEA could act as a preliminary certifying body for this purpose.

Given the current regulatory hurdles, one thing is clear: for the phage revolution to proceed, a more adaptable regulatory pipeline must be pursued in SEA (Jennes et al., 2017; Lin et al., 2017). Independent international organizations, such as Phages for Human Applications Group Europe (P.H.A.G.E), aim to do this by getting support from phage biologists, physicians, and policymakers in recognizing that phage therapy is a valid and promising option for treating bacterial infections in humans (http://www.p-h-a-g-e.org/, accessed on 20 December 2021). A similar organization or consortium can be made in the SEA to educate stakeholders, organize scientific fora, and exchange technical and regulatory experience on phage therapy applications in the region. Lastly, a research and biotechnology business model can be adapted to promote the sustainability of a phage revolution in SEA. Although phage therapy has weak support in terms of patent and market distribution, biotechnology companies and healthcare facilities could benefit from the phage industry by serving as niche or specialty providers (Fauconnier et al., 2020). These companies could use the phage specialization as leverage in creating spinoffs for phage therapy in SEA. To form these initiatives, the ASEAN could tap the WHO for resource capacity-building, and resource mobilization, especially for member states considered as low- to middle-income countries (LMICs; Fauconnier et al., 2020). The current business environment is not ideal for pharmaceutical companies to support phage formulations’ widespread production and marketing (Verbeken et al., 2014). Despite this drawbacks, we are optimistic that the utilization and ultimately market utilization of phage formulations and phage therapy will increase over the following years, with an estimated compound annual growth rate of 8.1% from 2021 to 2028 (Accurize Market Research, https://www.accurizemarketresearch.com/report/global-phage-therapy-market/, accessed on 15 July 2021). As of this writing, the Phage Directory (https://phage.directory/orgs, accessed 20 December 2021) and The Bacteriophage Ecology Group (http://companies.phage.org/, accessed on 20 December 2021) listed up to 60 biotechnology companies specializing in phages, none of which are SEA-based. This situation creates an opportunity for the SEA countries to establish their own regional spinoff company specializing in phages and phage therapy.

## Final Thoughts on Phage Revolution

Aside from being a global biodiversity hotspot, SEA can also be considered a global hotspot for antimicrobial resistance (Zellweger et al., 2017). Hence, the region must have effective therapeutic strategies to combat the growing resistance against antibiotics in the clinical setting. We view phage therapy as one of the most viable options in achieving this goal, given the opportunities present in the region. The historical data and the strategies we presented in this review point to the consideration of three things that would potentially spark the upcoming phage revolution in SEA: (1) increase the interest and knowledge of scientists in SEA about phages, (2) invest in virology facilities and systems, and (3) follow adaptable regulatory pipeline that will allow the use of phage therapy in clinics without hampering the quality of phage formulations.

The involvement of dedicated scientists is vital because they will become the pioneers of phage therapy in the region. They will also serve as the foundation in applying phage therapy knowledge in the clinics. Without these experts, the call for phage therapy in the region will be futile. To increase the interest in the field, the promotion of phage research by regional experts is critical. Creating a phage consortium or organization in SEA can help achieve this goal. As discussed in this review, the consortium could act as a technical expert for ASEAN-Approved Laboratories, a source of continuing education for the region, and provide an avenue where exchanges of ideas and practices about phage therapy could be discussed. As more experts in phages develop, we expect an increase in the number of scientific publications covering the understanding of phage biology, systematics, and anti-MDR organism potential, and eventually the rise of clinical trials focusing on the clinical applications of phages against MDR organisms. We also expect increased capacity-building activities to hone complementary skills for phage therapy implementation, such as electron microscopy, WGS, phage formulation techniques, phage administration, and bioprinting. The inaugural symposium on phage and phage-derived technologies in Singapore (2021), and the first virtual SEA phage workshop by Phages for Global Health in Malaysia (2022) are good starting points in increasing scientific interest on phage in the region. Meanwhile, to promote phage therapy appreciation among the public, citizen science programs that focus on accessible and appreciable activities involving phages can be done in schools following the model of the Science Education Alliance-Phage Hunters Advancing Genomics and Evolutionary Science (SEA-PHAGE, https://seaphages.org/, accessed last 20 December 2021). With educational activities, the concept of phage and phage therapy can be integrated within the public knowledge, hopefully making the phage revolution easier to implement and encourage.

As with all research-based endeavors, facilities and protocols are also essential to spark the phage revolution in SEA. But this aspect may not be an added economic burden to the region. As we have mentioned in this review, the creation of critical phage facilities like phage banks and phage testing centers could stem from existing facilities like the university-based phage collections or existing virological institutes, respectively. However, we believe that establishing a dedicated facility accessible to the SEA region would be more beneficial in creating a specific niche that could address concerns about phage therapy. The facility would be critical later on when regulations and standards on phage therapy have been created.

Lastly, following an adaptable model for the regulation and standardization of phage therapy would be a critical undertaking for all the countries within the ASEAN. We believe that the existing collaborations in the region regarding pharmaceutical and biological products regulations are vital starting points for creating adaptable guidelines applicable to SEA. These existing collaborations can be revisited and modified using the regulatory models from other countries; after these changes, regulatory agencies can accommodate more investigational and personalized therapeutic options like phage therapy. We accept that the regulatory aspect will take time. Still, the sooner the region starts its discussion on the matter, the sooner decision-makers can implement phage therapy for MDR organism-infected patients, who may have no other hope of survival other than exploring phage therapy.

In summary, a phage revolution that targets MDR organisms in SEA is an ambitious yet beneficial concept that should be opened to the scientists, clinical decision-makers, regulators, administrators, and other stakeholders of the region. In this review, we presented the details of preparing for this promising endeavor, starting from the basic understanding of the phages, the knowledge of phage therapy requirements, its historical use in the world and region, and the strategies to move forward. We hope that this review will open avenues for scientific and policy-based discussions on phage therapy and eventually lead the way to its fullest potential in combating MDR organisms in SEA.

## Author Contributions

MCa and RD conceptualized the topic and contents of the manuscript. MCa wrote and formatted the texts. MCr helped in formatting the texts. DdC-P, RR, and RD reviewed and provided expert opinion on the manuscript. All authors read and approved the final version of the manuscript.

## Funding

The authors received publication support from The Medical City.

## Conflict of Interest

The authors declare that the research was conducted in the absence of any commercial or financial relationships that could be construed as a potential conflict of interest.

## Publisher’s Note

All claims expressed in this article are solely those of the authors and do not necessarily represent those of their affiliated organizations, or those of the publisher, the editors and the reviewers. Any product that may be evaluated in this article, or claim that may be made by its manufacturer, is not guaranteed or endorsed by the publisher.
