# Supplementary material for: Phage Revolution Against Multidrug-Resistant Clinical Pathogens in Southeast Asia
Source: Front Microbiol. 2022 Jan 27;13:820572. doi: 10.3389/fmicb.2022.820572 (PMC8830912; doi:10.3389/fmicb.2022.820572)
Supplement: Supplementary file 1 [file Data_Sheet_1.docx]

Supplementary Table

**Supplementary Table 1.** Limitations and opportunities for advancements of traditional isolation and characterization methods for new phages with potential applications for therapy.

| **Methods** | **Limitations^1^** | **Opportunities for Advancement** |
| --- | --- | --- |
| **Phage isolation**  Enrichment procedure  Sample processing | Mostly described for isolating phages from the environment  Mostly done through centrifugation and filtration to obtain enough viral titer | Establishing protocols for phage isolation directly from clinical samples of the infected patients  Utilizing metagenomics to determine viral diversity in the sample coupled with chromatographic purification to obtain high quality, high titer preparations |
| **Phage Characterization**  Host range and sensitivity testing    Lytic growth  Phage taxonomy | Mostly described for testing activities against a defined set of hosts, physical and chemical conditions  Mostly done phenotypically by plaque formation testing  Mostly done using morphological data from electron microscopy studies | Establishing phagograms for newly discovered phages using standard set of quality control pathogens or pathogen strains coming directly from the patients  Utilizing whole genome sequencing for identifying lytic or lysogenic potential of the phage  Utilizing whole genome sequencing and metagenomics in characterizing phages based on reference genomes  Utilizing restriction fragment length polymorphism in pulse-field gel electrophoresis for classifying genetically similar phages  Utilizing sodium dodecyl sulfate polyacrylamide gel electrophoresis for classifying phages based on proteins |

^1^ Based on the methods described by Hyman (2019).

**Reference:**

Hyman P. (2019). Phages for Phage Therapy: Isolation, Characterization, and Host Range Breadth. *Pharmaceuticals (Basel), 12*(1):35. doi: 10.3390/ph12010035.
