# Supplementary material for: Phage Revolution Against Multidrug-Resistant Clinical Pathogens in Southeast Asia
Source: Front Microbiol. 2022 Jan 27;13:820572. doi: 10.3389/fmicb.2022.820572 (PMC8830912; doi:10.3389/fmicb.2022.820572)
Supplement: Supplementary file 2 [file Data_Sheet_2.docx]

Supplementary Figures


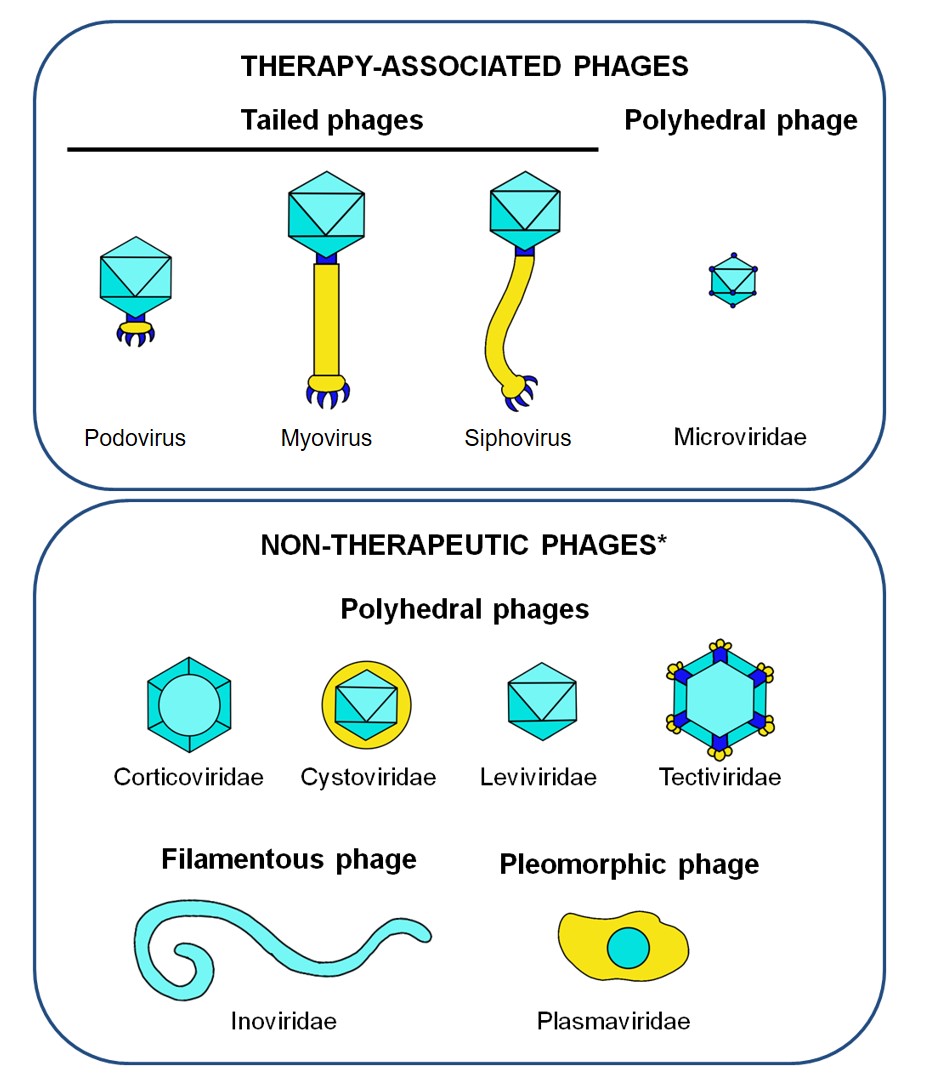


Supplementary Figure 1. Morphological classification of therapeutic and non-therapeutic phages. *Non-therapeutic phages refer to the phages that have not been used against clinical pathogens.


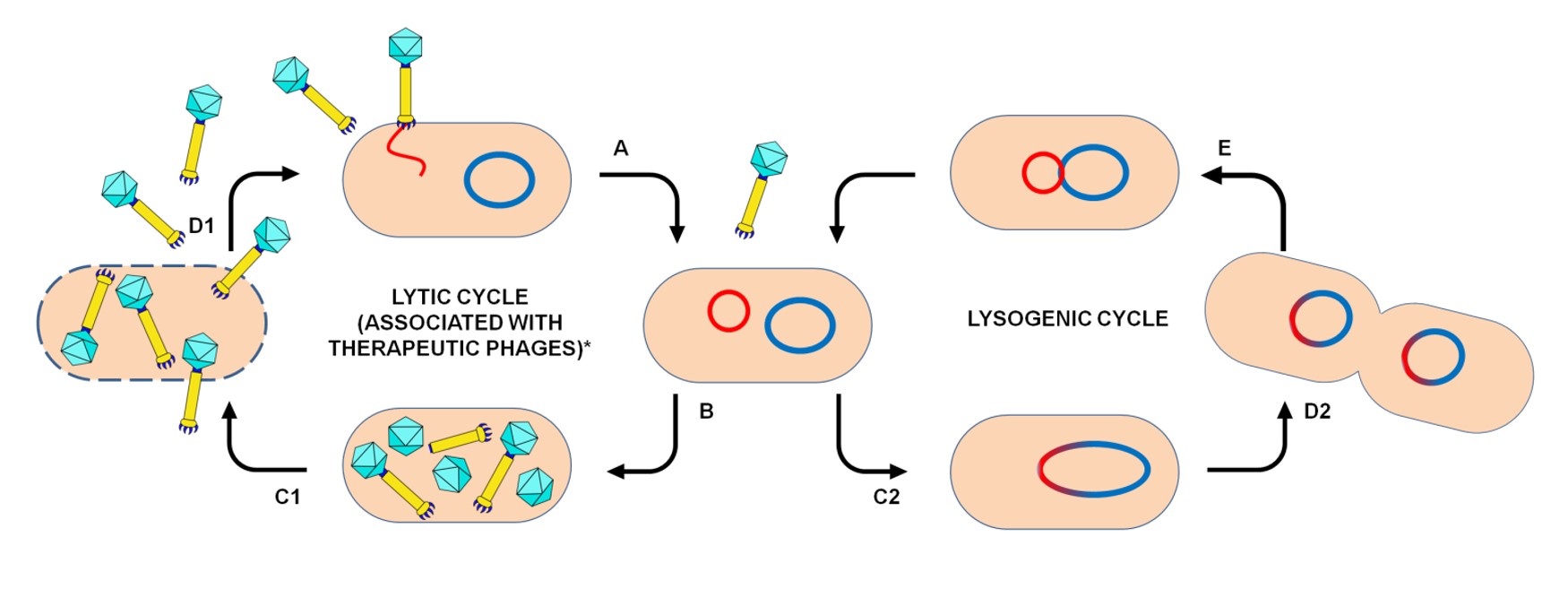


**Supplementary Figure 2.** General life cycle of phages. Lytic cycle involves the (A) attachment of the phage to its target bacteria and injection of its genomic material into the host, (B) circularization of phage genome, (C1) synthesis of page proteins and genome, and assembly into progeny virions inside the host, and (D1) lysis and release of progeny virions to infect other host populations. Lysogenic cycle still involves steps (A) and (B), but involves the (C2) formation of prophage through the combination of the phage genome with the bacterial chromosome, (D2) host cell and prophage reproduction, and (E) occasional prophage excision, recombination, and initiation of lytic cycle. Scientists also described other non-traditional life cycles (i.e., pseudolysogeny, chronic phage infection, and carrier state) in response to stresses such as host starvation, strain differences, quorum sensing, physiology, or changes in the environmental conditions. Only lytic phages are traditionally considered for phage therapy.


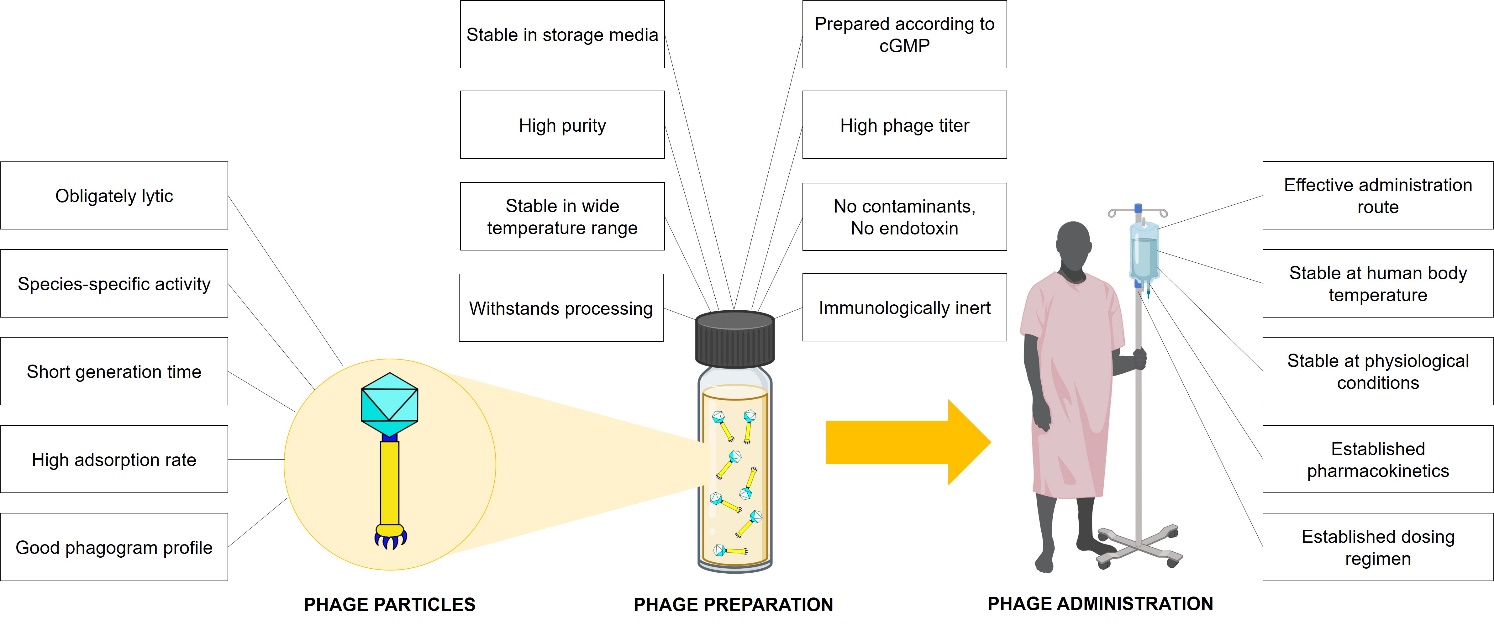


**Supplementary Figure 3.** Considerations in phage therapy. Different aspects of the phage characterization, preparation and administration should be considered for successful phage therapy applications.


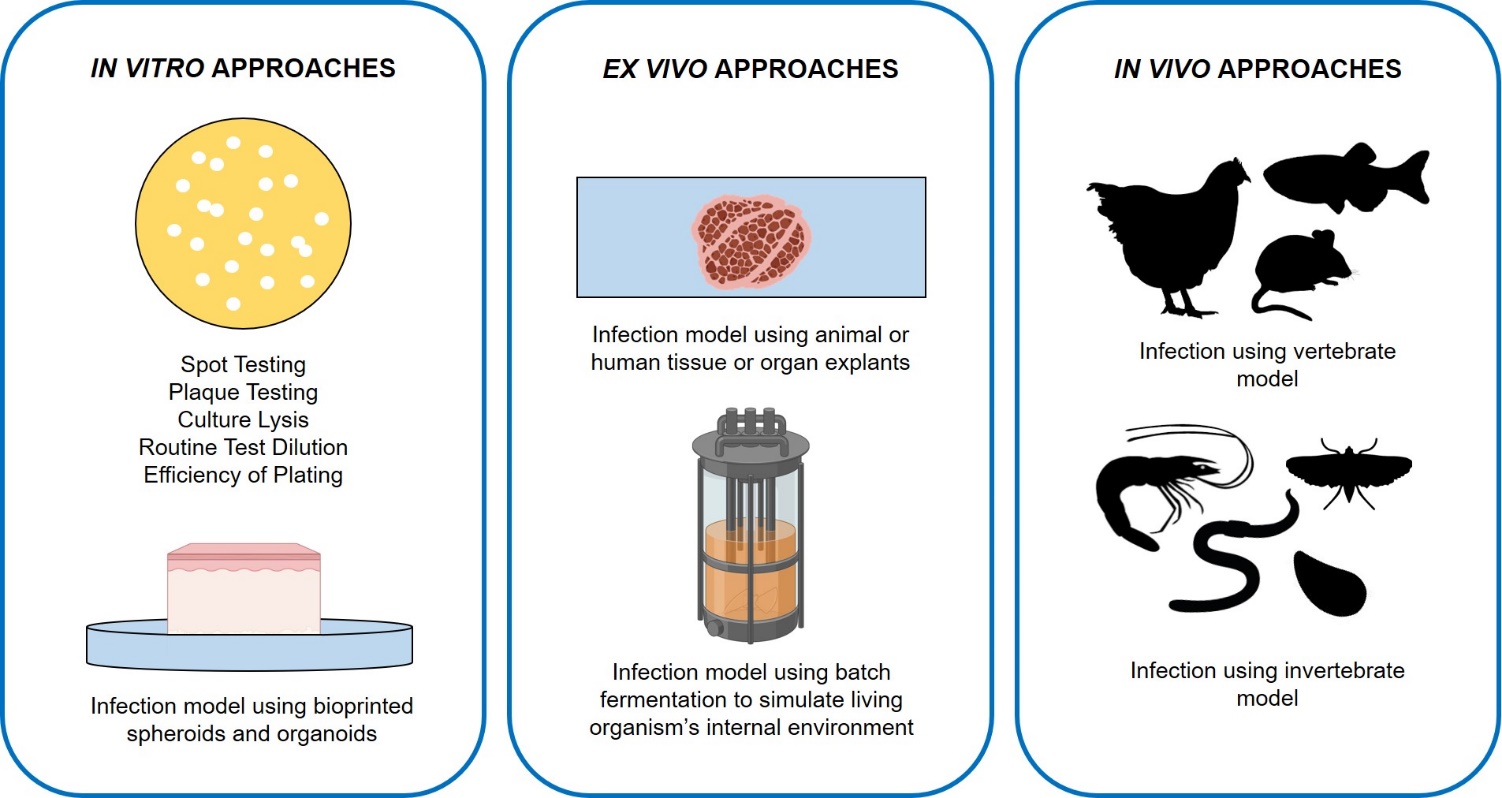


**Supplementary Figure 4.** Experimental approaches in studying phage efficacy and safety.
